# Comparison of Ensemble Techniques for Early Prediction of Alzhiemer Disease

**DOI:** 10.21203/rs.3.rs-5644910/v1

**Published:** 2024-12-23

**Authors:** Placida Orochi Orlunwo, Friday Eleonu Onuodu

**Affiliations:** Ignatius Ajuru University of Education; Ignatius Ajuru University of Education

**Keywords:** Machine learning, Ensemble, Adaptive Boost, Stacking, Gradient Boost, Bagging, Voting, Disease progression, Alzheimer’s disease

## Abstract

Alzheimer’s disease (AD) is a progressive neurological condition characterized by a loss in cognitive functions, with no disease-modifying medication now available. It is crucial for early detection and treatment of Alzheimer’s disease before clinical manifestation. The stage between cognitively healthy older persons and AD is known as mild cognitive impairment (MCI). To predict the transition from one-stage MCI to probable AD, five ensemble learning approach was used (Stacking, Gradient boost Bagging, Adaptive boost and Voting), an integrated model that combines not only cross-sectional neuroimaging biomarkers at baseline but also longitudinal cerebrospinal fluid (CSF) and cognitive performance biomarkers from the Alzheimer’s Disease Neuroimaging Initiative cohort (ADNI). The adaptive boost, stacking and bagging ensemble approach has shown potential to identify those at risk of developing Alzheimer’s disease, this would benefit them the most from a clinical trial or to use as a stratification approach inside clinical trials.

## 1. Introduction

The most widely recognized explanation of Alzheimer’s disease (AD) states that it begins as an abnormal amyloid buildup in the brain and progresses to dementia over a number of decades (Rudimar et al., 2018; Nicolai et al., 2020; Alvin et al.,2020; Sarah et al., 2020; Laurent et al., 2019). According to estimates from the Alzheimer Association as of March 2012, 5.4 million Americans have been diagnosed with AD, and more than 95% of them are 65 or older. Additionally, AD affects almost half of the population over the age of 85 (Modupe et al. 2022; Qi et al., 2014; Gesine and Peter, 2009; Jorge et al., 2018). The condition produces little strokes in the brain, which results in the slow cell death and nerve dysfunction in the brain. A person suffering from the condition may be unaware of the strokes because they occur without any perception (Malavika et al., 2020). The rate of advancement of Alzheimer’s disease (AD) differs amongst persons, thus rendering it impossible to gather precise projections of disease progression or time until specific medical outcomes for individual patients (Eric et al., 2017). This implies that effective prevention will necessitate predicting who will acquire AD decades before symptoms appear. As a result, there is a rising interest in establishing precise methods of identifying persons who are predisposed to developing symptomatic AD, in order to ensure they could be targeted for preventive interventions such as risk factor reduction, behavioural modification, or pharmacologic treatment. (Deborah and Sei, 2011; Lei et al., 2012).

Considering the disease cannot be cured, the acceptable treatment is limited to slow down the progression of the ailment. Early disease detection will be beneficial for the doctor, the patient’s family, other close friends, etc. As a result, machine learning approaches are utilized to detect the disease early. In order to achieve the finest degree of accurateness, five strategies of ensemble methods are applied. Gradient boost, Stacking, Bagging, Voting and Adaptive boost Classifier are the approaches used. Using Python script for implementation, the most suitable and accurate model can possibly be recognized.

Prior models for Alzheimer’s disease risk prediction are often based on preset health profile variables such as sociodemographic (age, gender, education), lifestyle (physical activity), midlife health risk factors (systolic blood pressure, BMI, and total cholesterol level), and cognitive profiles (Alexander et al.,2019; Ji et al., 2020). Aside from identifying Alzheimer’s disease, forecasting the severity of cognitive impairment is a clinically significant challenge. Previous research has demonstrated that markers of primary AD pathology, neurodegeneration (structural MRI, FDG-PET), or biomarker combinations can predict whether a person would progress from moderate cognitive impairment (MCI) to AD dementia (Nicolai et al., 2019; Longfei et al., 2020; Xiaoying et al., 2017; Ting et al., 2019; Gang et al., 2011; Sarah et al., 2020). The moment these alterations start to take place and the point at which they may be distinguished from normal aging are, however, not well understood. This question is critical for establishing measurements that may be more sensitive to recognizing persons in the preclinical stage of the disease, as well as for therapeutic implications. As more effective pharmacological therapies for Alzheimer’s disease become available, it will become increasingly vital to develop and deploy preventive techniques to identify persons with preclinical dementia earlier in the illness’s natural course. Artificial intelligence-based 18F-FDG-PET analysis, including machine learning and deep learning, has gradually entered mainstream computing (Charles et al., 2000; Janani et al.,2021; Mohamed et al., 2018; Aliaa et al., 2022).

Advances in artificial intelligence, particularly in the field of machine learning, pose novel challenges as a result of the merging of computer science and biomedical sciences (Pushkar, 2019). The topic of big data with high data dimensions is being researched in the field of medicine, particularly with regard to magnetic resonance imaging (MRI) images. As the Internet and databases advance, big data continues to grow and advance tremendously. This is especially true in the case of medical large data and imagery. As a result, the issue of increasing data demonstrates the concept and potential of big data (Firouzeh et al., 2019).

## 2. Related Study

Nria et al. (2020) described the variations in the short-term temporal network dynamics of undirected and weighted whole-brain functional connectivity between healthy aging persons and people with mild cognitive impairment (MCI). The Network Change Point Detection technique was used to identify major change points in the resting-state fMRI register, and the fluctuations in the topological features of the sub-networks between significant change points were investigated. Ji et al. (2020) used large-scale administrative health data from the Korean National Health Insurance Service database between 2002 and 2010 to test the feasibility of using trained and validated random forest, support vector machine, and logistic regression machine learning to predict AD incidents in 1, 2, 3, and 4 subsequent years.

Shweta et al. (2020) used machine learning methods to identify dementia in its early stages, including Random Forest Classifier, (SVM), Decision Tree Classifier, Extra Tree Classifier, Neighbours Classifier, and Logistic Regression. Gender, age, education, MMSE, CDR, ASF, Handedness, and the number of hospital visits of patients classified as demented or non-demented make up the data for inquiry. Gopi et al. (2020) designed a decision tree model to forecast Alzheimer’s disease (AD) in the future. In this study, demographic variables from 150 participants and 373 MRI sessions were evaluated. To perform predictive analysis on Alzheimer’s disease patients, pruned decision trees (J48) were used.

Weiming et al. (2020) created an ELM-based grading approach to efficiently fuse multimodal data and predict MCI-to-AD conversion. First, features from magnetic resonance (MR) images were retrieved, and then valuable features were chosen using a feature selection method. subsequently these grading scores from several modalities were entered into a classifier to distinguish participants with progressing MCI from those with stable MCI. The research by Matoug et al. (2019) described a pseudo-automatic method that reads volumetric MRI, extracts the middle slices of the brain region, performs segmentation to find the region of the brain’s ventricle, generates a feature vector that describes this region, creates a SQL database that contains the generated data, and then categorizes the images using the extracted features.

Xiaojing et al. (2016) developed a machine learning method to distinguish patients with AD or moderate cognitive impairment (MCI) from healthy elderly and to predict AD conversion in MCI patients by computing and evaluating localized morphological variations in the brain between groups. Asymmetric diffeomorphic registration was used to calculate the distance between each pair of subjects, which was then followed by an embedding algorithm and a learning approach for classification.

## 3. Proposed Methodology

An all-encompassing meta-approach to machine learning called ensemble learning aims to improve predictive performance by pooling predictions from many models called base leaners. Whilst one can create an apparently infinite number of ensembles to tackle model prediction, three strategies dominate the field of ensemble learning which are Boosting, Stacking and Bagging. Sequential and parallel ensemble techniques are the two primary kinds of ensemble methods.

**Sequential ensemble**. approaches produce base learners in a sequential order. The sequential production of basic learners fosters dependability among the base learners. The model’s performance is then improved by giving bigger weights to previously misrepresented learners.

**Parallel ensemble** approaches generate base learners in a parallel fashion, such as random forest. Parallel techniques make use of the parallel generation of base learners to develop independence among the base learners. The independence of base learners considerably lowers the inaccuracy caused by the use of averages.

In base learning, the majority of ensemble strategies use a single algorithm, resulting in homogeneity across all base learners. Homogenous base learners are base learners of the same type with similar characteristics. Heterogeneous base learners, resulting in heterogeneous ensembles.

**Figure 1:**
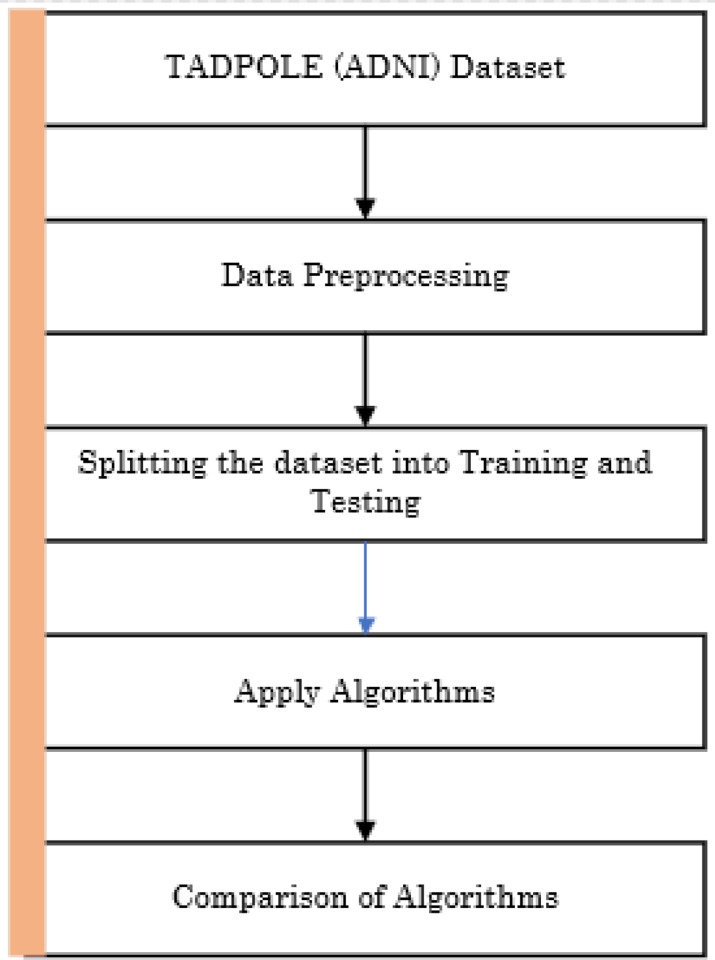
Proposed process framework

In the healthcare sector, machine learning has an enormous impact. The healthcare area has an immense quantity of datasets to design an advanced and scientific way to diagnose the disease at an early stage. As a result, some machine learning algorithms are employed for predicting symptoms and choosing the top precision supplier among all of these approaches. The proposed strategy as in Fig-1 employs the ensemble technique for forecasting the five phases of Alzheimer’s disease in advance. Adaptive Boost, Gradient Boost, Stacking, Voting and Bagging are the algorithms used and the implementation is done in Python programming language.

### 3.1 Data Collection

Data used in the preparation of this article were obtained from the Alzheimer’s Disease Neuroimaging Initiative (ADNI) database (adni.loni.usc.edu). The ADNI was launched in 2003 as a public-private partnership, led by Principal Investigator Michael W. Weiner, MD. The primary goal of ADNI has been to test whether serial magnetic resonance imaging (MRI), positron emission tomography (PET), other biological markers, and clinical and neuropsychological assessment can be combined to measure the progression of mild cognitive impairment (MCI) and early Alzheimer’s disease (AD). Table 1 shows the specifics of the dataset used in this investigation. The baseline combined dataset included 12741 occurrences, 3821 of which were Mild Cognitive Impairment - CN, 2319 Early Mild Cognitive Impairment - EMCI, 389 Significant Memory Concern - SMC, 4644 Late Mild Cognitive Impairment - LMCI and 1568 participants recorded to have developed Alzheimer Disease - AD. The dataset includes people ranging in age from 55 to 96, both male and female, as well as many other characteristics that can be utilized to train and execute algorithms to detect the impact of Alzheimer’s disease.

### 3.2 Data Preprocessing

All modifications to the raw data before they are delivered to the machine learning algorithm is referred to as data preprocessing. Poor classification performance is likely to result from training a model on an unprocessed dataset. Preprocessing is essential for accelerating training methods like clustering and scaling. Real-world data is frequently inaccurate and lacking in specific behaviours or trends. It is also frequently inconsistent and incomplete. A tried-and-true technique for tackling such problems is data preprocessing. The dataset was first examined to see if there were any categorical values, and it did contain a few of them. The gender and marital status attribute columns are among them and are changed into the numbers 0–1 and 1–4. In order to better comprehend them, we have examined the correlation between qualities using the “correlation matrix” function based on group attributes and plotted them. The dataset is then examined for any null or missing values.

### 3.3 Splitting the dataset into Training and Testing

Based on the holdout approach, the dataset is split into a training set and a testing set (Bookheimer et al., 2000). According to several researchers in the literature, 80% of the dataset (used as a testing set) is sufficient to produce accurate results (Huang et al., 2017; Seijo-Pardo et al., 2017). As a result, the prediction model was created using 80% and 20% size of testing and training set required to achieve the best results.

### 3.4 Apply Algorithm

In the present investigation, a comparative analysis with well-known methods has been done using the Alzheimer’s Disease Neuroimaging Initiative (ADNI) database (adni.loni.usc.edu). Bagging, Stacking, Voting, Adaptive Boost, and Gradient Boost are the ensemble methods used for early AD detection. The rest of this subsection presents a summary of the selected techniques.

#### Boosting

The term “boosting” refers to a class of algorithms that can convert weak models into strong models. Boosting works by placing weak learners in succession such that weak learners can learn from the next learner in the sequence, resulting in improved prediction models. Gradient boosting, Adaptive Boosting (AdaBoost), and XGBoost (Extreme Gradient Boosting) are all examples of boosting, however AdaBoost and Gradient Boost has been chosen for the study.

#### Adaptive Boost

AdaBoost employs weak learners in the form of decision trees, which typically feature one split, also known as decision stumps. AdaBoost’s fundamental deciding stump is made up of inputs with equal weights.

#### Gradient Boost

Gradient boosting increases the ensemble’s predictor count incrementally, with earlier predictors correcting subsequent ones in order to improve the precision of the model. In order to mitigate the consequences of previous prediction failures, new predictors are fitted. The gradient booster uses the gradient of descent to find and fix predicted errors made by students.

#### Bagging

This strategy, also known as “Bootstrap Aggregating,” summarizes the key elements of this approach. Bootstrapping and aggregation are the two methods of bagging.

Bootstrapping is a sampling strategy where samples are taken utilizing the method of substitution from the entire sample (set). The sampling with the substitution method aids in the randomization of the selection process. The process is finished by applying the base learning algorithm to the samples.

Aggregation is used to include all potential outcomes of the prediction and randomize the result. Predictions made without aggregation won’t be accurate because all possible outcomes won’t be taken into account. As a result, the aggregate is based either on all of the results from the predictive models or on the probability bootstrapping techniques.

#### Voting

Voting ensembles are a subset of ensemble techniques. They use several models to train on the dataset and provide predictions because they are one of the ensemble methods. The entire dataset is fed to several models of various machine learning algorithms in Voting Classifiers, and each algorithm makes predictions after being trained on the data. The most common approach is used to obtain the final prediction from the model after all of the models have predicted the sample data. The category that the various algorithms predicted most accurately in this case will be treated as the model’s final prediction.

#### Stacking

Also known as stacked generalization, is an ensemble method that combines models using another machine learning algorithm. The basic idea is to train machine learning algorithms on training datasets and then use these models to generate new datasets. The new dataset is then fed into the combiner machine learning algorithm.

### 3.5 Model Evaluation

Fig-5 is confusion matrix representing adaptive boost method with the various labels encoded (CN-0, SMC-1, EMCI-2, LMCI-3, and AD-4). Table 2 is a statistical breakdown of the data in Fig-5, Table 3 displays the key performance parameters Precision, Recall, and F1 scores for five phases of Alzheimer disease and Table 4 shows the performance of the categorization models on the test data.

## 4. Result and Discussion

In this study, every one of the classification models is trained using 10 folds cross-validation on the training dataset. In order to prevent model overfitting, cross-validation has been carried out with the training dataset. The learners’ effectiveness has been assessed using an unobserved test dataset. Table 4 shows the categorization performance of the models across both the training and testing periods.

**Figure 4:**
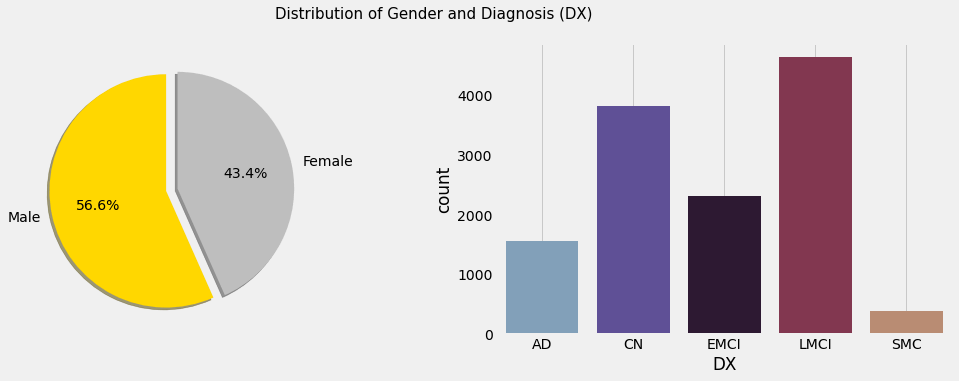
Gender and diagnosis statistics

**Figure 5:**
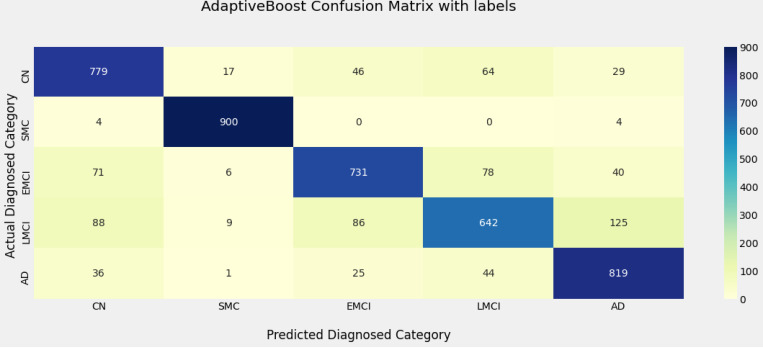
Confusion matrix of Adaptive boost

According to the results analysis in Fig-4, three ensemble approaches, Stacking, Adaboost, and Bagging beat the other two classifiers and provided an accuracy of 97% over the test time. Voting and GBoost have reasonable accuracies of 81% and 95%, respectively. It is also worth noting that the results obtained on test data are very similar to the findings obtained throughout the cross-validation period. This demonstrates that the created models were not overfitted during the training phase. AdaBoost ensemble model accurately categorized the majority of the unseen instances of the CN, SMC, EMCI, LMCI and AD classes, with class precisions [80%, 96%, 82%, 78%, 81%], class recall [83%, 99%, 79%, 68%, 89%], F1 Score [81%, 98%, 81%, 72%, 84%] respectively. The SMC class findings suggest that testing instances have been misclassified. Whilst in Fig-5, 17 out of 933 instances of the SMC class were misclassified as the CN class. This is due to the fact that patients in the SMC class have clinical assessment characteristics that are extremely comparable to those found in the CN class. Furthermore, 78 of the 828 LMCI cases have been incorrectly classified as EMCI. This is due to the characteristic values of the EMCI and LMCI classes intersect.

## 5. Conclusion

This research compares and evaluates recent work in the prognosis and prediction of Alzheimer’s disease using ensemble learning approaches. Obviously, significant machine learning advances have been reported that cover the categories of data employed and the effectiveness of algorithms based on machine learning for identifying the earliest stages of Alzheimer’s. It has become evident that machine learning improves the precision of forecasts, particularly when compared to traditional statistical techniques. There are a number of improvements to our dataset and methodology that are important steps for future research. Here, we limited ourselves to modelling 14 biomarkers that are commonly measured in AD clinical trials. We had excluded some interesting ones due to high number of null values; whilst over sampling is good to balance up data this can equally lead to model overfitting, in future other methods would be employed to handle the situation of data imbalance.

## Figures and Tables

**Figure 2:**
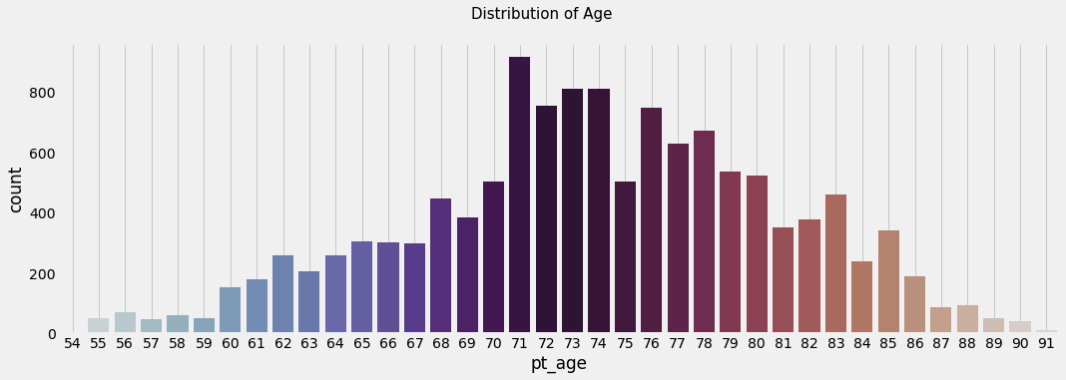
Age statistics

**Figure 3:**
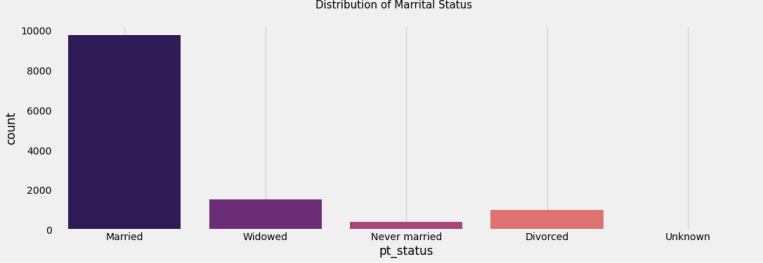
Marital statistics

**Table 1:** Dataset statistics

Alzheimer Stages	No. of Records
Mild Cognitive Impairment - CN	3821
Significant Memory Concern - SMC	389
Early Mild Cognitive Impairment - EMCI	2319
Late Mild Cognitive Impairment - LMCI	4644
Alzheimer Disease - AD	1568
Total	12741

**Table 2:** Statistics of Adaboost confusion matrix

	CN	SMC	EMCI	LMCI	AD	TOTAL
**CN**	779	17	46	64	29	**935**
**SMC**	4	900	0	0	4	**908**
**EMCI**	71	6	731	78	40	**926**
**LMCI**	88	9	86	642	125	**950**
**AD**	36	1	25	44	819	**925**
**TOTAL**	**978**	**933**	**888**	**828**	**1017**	**4644**

**Table 3:** Performance evaluation using Recall, F1 Score and Precision.

Methodology	PRECISION					RECALL					F1 SCORE				
	CN	SMC	EMCI	LMCI	AD	CN	SMC	EMCI	LMCI	AD	CN	SMC	EMCI	LMCI	AD
Bagging	80	96	81	77	78	82	99	81	63	88	81	98	81	69	83
Gboost	75	92	82	75	75	79	98	79	60	85	77	95	80	66	80
Adaboost	80	96	82	78	81	83	99	79	68	89	81	98	81	72	84
Voting	75	94	81	80	77	84	99	80	55	89	79	97	81	65	83
Stacking	79	99	85	74	86	84	99	80	74	86	81	99	82	74	86

**Table 4:** Classification Performance

General Accuracy (%) on Train and Test Set Data		
Methodology	Train Set	Test Set
GBoost	88	95
Voting	92	81
Stacking	93	97
Bagging	93	97
AdaBoost	93	97
